# Differentiating Upper Tract Urothelial Carcinoma with Synchronous or Metachronous Bladder Cancer

**DOI:** 10.3390/cimb48040345

**Published:** 2026-03-26

**Authors:** Sara Meireles, Carolina Dias, Ana Marques, João Silva, Luís Costa, José Manuel Lopes, Paula Soares

**Affiliations:** 1 Institute for Research and Innovation in Health (i3S), University of Porto, Rua Alfredo Allen 208, 4200-135 Porto, Portugal; 2 Institute of Molecular Pathology and Immunology of the University of Porto (IPATIMUP), Rua Júlio Amaral de Carvalho 45, 4200-135 Porto, Portugal; 3 Faculty of Medicine, University of Porto, Alameda Professor Hernâni Monteiro, 4200-319 Porto, Portugal; 4 Medical Oncology Department, Centro Hospitalar Universitário de São João (CHUSJ), Alameda Professor Hernâni Monteiro, 4200-319 Porto, Portugal; 5 Pathology Department, Centro Hospitalar Universitário de São João (CHUSJ), Alameda Professor Hernâni Monteiro, 4200-319 Porto, Portugal; 6 Urology Department, Centro Hospitalar Universitário de São João (CHUSJ), Alameda Professor Hernâni Monteiro, 4200-319 Porto, Portugal; 7 Medical Oncology Department, Centro Hospitalar Universitário Lisboa Norte, Avenida Professor Egas Moniz MB, 1649-028 Lisboa, Portugal; 8 Institute of Molecular Medicine João Lobo Antunes, Faculty of Medicine, University of Lisbon, Avenida Professor Egas Moniz MB, 1649-028 Lisboa, Portugal

**Keywords:** upper tract urothelial carcinoma, bladder cancer, synchronous, metachronous, prognosis

## Abstract

The features of patients with multiple urothelial tumors remain to be elucidated. We intend to differentiate primary upper tract urothelial carcinoma with synchronous urothelial bladder cancer (UTUC + sUBC) and UTUC with metachronous UBC (UTUC + mUBC) cases to determine whether these temporal patterns reflect biologically distinct processes. A subgroup analysis of a retrospective cohort of UTUC (*n* = 114) was performed comparing UTUC + sUBC (*n* = 14) with UTUC + mUBC (*n* = 29). IHC expression of cytokeratin 5/6 (CK5/6), CK20, GATA3, and p53 was evaluated to assess relevant subtypes. Genetic characterization comprised *TERTp*, *FGFR3*, *RAS*, and *TP53* status. Kaplan–Meier analyses estimated the progression-free survival (PFS) and overall survival (OS) of both UTUC subgroups, and the log-rank test was used to assess differences between subgroups. Our study reveals no significant differences in phenotype or genomic profile between synchronous and metachronous UTUC-UBC cases (*p* > 0.05). Nevertheless, patients with synchronous UBC revealed significantly worse outcomes in PFS (2y-PFS 23.1% vs. 52.1%, *p* = 0.029) and OS (2y-OS 40.4% vs. 84.4%, *p* = 0.016) than those with metachronous disease. These discrepancies could arise from as yet-uncharacterized molecular features or microenvironmental influences.

## 1. Introduction

Urothelial carcinomas are the most common histologic subtype of all genitourinary malignancies [1]. The potential to develop multiple foci throughout the urinary tract allows the opportunity to study their temporal and anatomic disease biology [2].

Patients diagnosed with primary upper tract urothelial carcinoma (UTUC) have the greatest risk of developing future urothelial bladder cancer (UBC) [3,4]. Between 22% and 47% of UTUC patients who undergo radical nephroureterectomy (RNU) have a metachronous diagnosis of UBC within the first two years [5,6,7]. The prevalence of synchronous UBC in UTUC patients is approximately 17% [8].

The timing of bladder cancer occurrence may reflect distinct biological pathways and could have implications for prognosis and surveillance strategies. One main hypothesis suggests that these bladder tumors may arise from intraluminal seeding of tumor cells from the UTUC [9], whereas an alternative explanation is a pan-urothelial field effect, in which the entire urothelium undergoes molecular alterations predisposing one to independent tumor development [10].

Molecular studies indicate that most UTUC-UBC tumor pairs share a clonal origin [11], although complete genomic congruence is rare [12]. Sometimes UBC can develop from a standard progenitor clone that precedes the initial UTUC diagnosis [11]. Therefore, molecular analysis may reveal additional genomic alterations in the UBC along with overlapping mutations. Identifying these divergent alterations could help refine risk stratification and personalize therapeutic approaches [12].

To date, there is limited research on biomarker expression in primary UTUC with associated UBC. Our prior work has shown that UTUC-only and UTUC-UBC patients display similar molecular and pathological features [13].

Here, we aim to differentiate primary UTUC with synchronous UBC (UTUC + sUBC) and UTUC with metachronous UBC (UTUC + mUBC) cases to determine whether these temporal patterns reflect distinct molecular phenotypes and independent biological processes.

## 2. Materials and Methods

### 2.1. Study Design

The authors compiled a retrospective cohort of patients diagnosed with UTUC who underwent radical nephroureterectomy resection or a kidney-sparing approach at our institution between January 2009 and December 2019. Patients with a prior diagnosis of UBC or those who received neoadjuvant or adjuvant systemic treatment were excluded. Cases of UTUC with synchronous (within six months) [11] or metachronous UBC diagnosis were selected for analysis. The cases were categorized into two subgroups: (1) UTUC with synchronous UBC (UTUC + sUBC) (*n* = 14); (2) UTUC with metachronous UBC (UTUC + mUBC; *n* = 29). Patients presenting with metastasis at diagnosis were excluded from the survival analysis.

Baseline demographic, clinicopathological, and outcome data were collected in a database following a comprehensive review of electronic medical records.

### 2.2. Immunohistochemical and Genomic Characterization

All archived formalin-fixed, paraffin-embedded (FFPE) tumor specimens from UTUC patients who met the inclusion criteria were retrieved and analyzed. Representative hematoxylin and eosin (H&E)-stained sections were reviewed by an expert genitourinary pathologist, who was blinded to the clinical outcomes and the subgroup categorization (synchronous vs. metachronous) of the patients.

Tumor subtypes were determined by immunohistochemical (IHC) analysis using antibodies against cytokeratin 5/6 (CK5/6), CK20, GATA3, and p53. IHC staining was performed using the UltraVision^TM^ Quanto Detection System HRP (REF: TL-125-QHL, Thermo Scientific, Waltham, MA, USA). GATA3 immunostaining (clone L50-823, ready-to-use; Master inVitro Diagnóstica, Sevilla, Spain) was conducted on a Ventana Benchmark XT automated staining platform (Ventana Medical Systems, Tucson, AZ, USA).

Molecular profiling included assessment of *TERT* promoter (*TERTp*), *FGFR3*, *RAS*, and *TP53* alterations. Hotspot mutations in the *TERT* promoter (positions −124 and −146 relative to the transcription start site, NM_198253) and in *FGFR3* exon 7 (codons 248 and 249, NM_000142) were analyzed using quantitative real-time PCR (QuantStudio^TM^ 5 Real-Time PCR System, Applied Biosystems, Waltham, MA, USA) according to the Uromonitor^®^ kit protocol (U-monitor, Porto, Portugal). Mutations in *TP53* exons 5–9 (NM_000546), as well as in *HRAS* (NM_005343) and *KRAS* (NM_004985) codons 12, 13, and 61, were assessed by Sanger sequencing. For *NRAS* (NM_002524), only codon 61 was analyzed.

All detailed experimental procedures are described in Supplementary File S1. The patient selection process and study design to molecular characterization are summarized in Figure S1 (Supplementary File S2).

### 2.3. Outcomes and Statistical Analysis

The Statistical Package for Social Sciences software (SPSS, IBM Corp., Chicago, IL, USA), version 28.0, was used for all statistical analyses.

Categorical variables were expressed as frequencies and percentages and compared using the Pearson’s Chi-square test or Fisher’s exact test, as appropriate (when expected frequencies were less than 5). Continuous variables were assessed for normality using the Shapiro–Wilk test. As the distribution was non-normal, comparisons between groups were performed using the Mann–Whitney U test.

Kaplan–Meier analyses was used to estimate the progression-free survival (PFS) and overall survival (OS) of both UTUC subgroups, and the log-rank test was used to assess differences between subgroups.

Overall survival and progression-free survival were defined as the interval between the date of diagnosis and the date of death/disease progression or last follow-up. Disease progression was characterized by distant metastases and newly diagnosed local disease (upper urothelium). Bladder recurrences were considered progression events if they occurred after the initial diagnosis of a bladder tumor (synchronous or metachronous).

A sensitivity analysis of PFS was performed by excluding all intravesical events from the PFS calculation to mitigate potential circularity. Additionally, a 6-month landmark analysis was conducted to account for potential immortality time bias. For this analysis, only patients who remained alive and event-free at 6 months were included, and follow-up time was recalculated starting from the 6-month landmark point.

A *p*-value < 0.05 was considered statistically significant. No formal adjustment for multiple comparisons was applied due to the exploratory nature of this study; therefore, *p*-values should be interpreted with caution.

## 3. Results

### 3.1. Clinicopathological Characteristics of UTUC Patients with Synchronous or Metachronous UBC

A total of 43 patients with primary UTUC had a synchronous (*n* = 14) or metachronous UBC (*n* = 29) diagnosis. The median age at diagnosis was 77 years (interquartile range, IQR: 67–82), and 69.8% (*n* = 30) of the patients were male. Most patients had stage ≥ II (69%, *n* = 29) and a high-grade tumor (93%, *n* = 40). Both subgroups were comparable in terms of demographic and clinicopathological variables (Supplementary Table S1).

### 3.2. Immunohistochemical and Mutational Status Rates in UTUC + sUTUC vs. UTUC + mUBC Subgroups

Immunohistochemical and mutational analysis were used to assess the molecular differences between UTUC + sUBC and UTUC + mUBC subgroups.

UTUC + sUBC and UTUC + mUBC did not differ significantly in mutational status or in terms of luminal and basal-like stratification (all *p*-values > 0.05). Detailed IHC and mutational profiling in both subgroups are summarized in Table 1.

### 3.3. Survival Analysis of UTUC + sUTUC and UTUC + mUBC Subgroups

**Figure 1:**
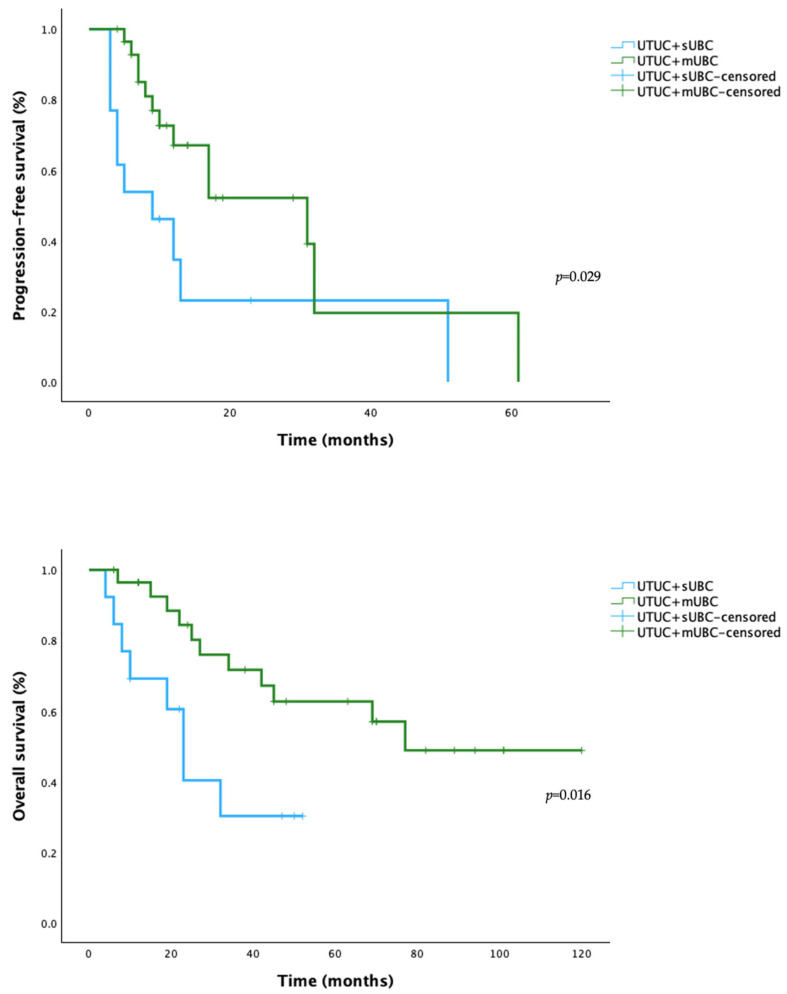
Kaplan–Meier curves comparing progression-free survival (PFS) and overall survival (OS) between patients with UTUC + sUBC (blue line) and those with UTUC + mUBC (green line) patients. One patient was excluded from the survival analysis due to the presence of metastasis at diagnosis. Abbreviations: UBC—urothelial bladder cancer; UTUC—upper tract urothelial carcinoma; UTUC + mUBC—UTUC with metachronous UBC; UTUC + sUBC—UTUC with synchronous UBC. Log-rank test, statistical significance *p*-value < 0.05.

The median follow-up time was 32 months (IQR: 15–69). The progression-free survival (2y-PFS 23.1% vs. 52.1%, *p* = 0.029) and OS (2y-OS 40.4% vs. 84.4%, *p* = 0.016) were consistently worse in the UTUC + sUBC cohort than in the UTUC + mUBC subgroup (Figure 1). The median of PFS for UTUC + sUBC and UTUC + mUBC was 9 months (95% CI 0.95–17.1 *p* = 0.029) and 31 months (95% CI 11.7–50.3 *p* = 0.029), respectively. The survival advantage for the synchronous group remained statistically significant even when PFS was performed by excluding all intravesical events (*p* = 0.04)—Supplementary Figure S2.

Regarding OS, the median was 23 months (95% CI 17.2–28.8 *p* = 0.016) and 77 months (95% CI 60.9–96.9 *p* = 0.016) for the same subgroups.

The results were further validated by a 6-month landmark sensitivity analysis, which maintained statistical significance for both PFS (*p* = 0.047) and OS (*p* = 0.038)—Supplementary Table S2.

## 4. Discussion

The clinical and molecular features of patients with multiple urothelial carcinomas remain incompletely defined. Cases of UTUC and UBC occurring in the same individual allow for the exploration of both the temporal and spatial dimensions of urothelial carcinogenesis.

Our previous research revealed that primary UTUC-only and paired UTUC-UBC express comparable clinicopathologic features and similar molecular profiles [13,14], suggesting shared tumor biology irrespective of secondary bladder involvement. Additionally, the occurrence of UBC did not correlate with worse prognosis in UTUC patients, even after stratification by molecular and luminal–basal subtypes [13,14]. These findings reflect differences in clinical behavior between UTUC and its bladder counterpart. Although both tumors affect urothelial tissues, potentially distinct oncogenic pathways drive their carcinogenesis [15,16].

Our current study revealed no significant differences in phenotypic or genomic profiles when comparing UTUC patients with synchronous (sUBC) versus metachronous (mUBC) bladder tumors.

The underlying mechanisms of concomitant or subsequent UBC in patients with UTUC remain a topic of ongoing investigation. Identifying a prevailing hypothesis to address this issue has been challenging because both current theories may coexist. The monoclonality of multifocal urothelial tumors is supported by several studies [17], including evidence of high-grade concordance (73%) between paired UTUC and UBC [18] and consistent genomic profiles across multifocal lesions [19,20]. In our series, the uniform biomarker expression across all UTUC subgroups further highlights the monoclonality hypothesis, regardless of UBC occurrence or timing. However, these findings do not allow for definitive conclusions about the clonal origin and comparative intrinsic aggressiveness between both entities.

Conversely, the uncertainty of whether metachronous bladder cancers are truly second primary tumors or intravesical recurrence is still up for debate.

Emerging molecular data suggest that even metachronous cancers are considerable related to the original tumor’s molecular subtype, irrespective of their anatomic origin or chronologic growth [21]. This perspective sustains the concept of intraluminal tumor cell seeding rather than the emergence of an entirely new malignancy.

Prior studies on unrelated non-muscle invasive bladder cancer (NMIBC) disclosed mutational patterns like those in early-stage UTUC (pTa, pT1), suggesting a broader biological overlap. Our previous molecular analysis of 125 primary non-muscle invasive bladder cancers also revealed a high genetic similarity among primary UTUC and an unrelated primary UBC [13].

Despite molecular homogeneity, our survival analysis identified significantly worse PFS and OS in patients with UTUC + sUBC compared to those with UTUC + mUBC. This disparity was observed in absence of clinicopathologic or genomic differences and after excluding patients who received neoadjuvant or adjuvant therapy. It is still uncertain whether UTUC + sUBC are linked to a greater tumor burden or enhanced aggressiveness, potentially resulting in higher recurrence rates. These outcome divergences may be assigned to yet-unidentified prognostic biomarkers and raise the possibility of microenvironmental influences. Moreover, the temporal sequence of tumor appearance may not reflect the underlying clonal evolution [11] and inconsistent definitions for synchronous versus metachronous disease further hinder cross-cohort comparisons. Besides, different methods have been employed throughout each series, thwarting these conclusions [11].

Although our findings are suggestive of a common biological background, the potential contribution of field cancerization to unrelated tumors in the upper and lower urinary tracts cannot be excluded. Additionally, in the absence of paired clonal analysis between UTUC and UBC, the possibility of independent primary tumors (oligoclonality) or intra-luminal seeding remains to be fully elucidated.

Characterizing the tumor immune microenvironment is essential to clarify the effect of immune responses in the biology of multifocal urothelial carcinomas and the potential implications for immunotherapy.

Finally, some limitations of this study should be acknowledged. Beyond this study’s retrospective and exploratory design and the limited statistical power of the relatively small patient cohort, our genomic analysis was restricted to selected hotspot mutations. Furthermore, the lack of a universal consensus regarding the synchronicity threshold remains a significant challenge that warrants standardization in future multicenter trials. Lastly, integrating data on the prevalence of patients with Lynch syndrome could provide important insights into the survival outcomes of UTUC.

Further prospective studies with larger cohorts and extended follow-up are required to validate these observations. Moreover, whole-exome sequencing or spatial transcriptomics and deeper genetic factors are needed to explore the key drivers of urothelial disease. Such high-resolution approaches will be essential to refine risk stratification models and better understand the biological complexity of multifocal urothelial cancer.

## 5. Conclusions

UTUC exhibits distinct anatomical and biological features associated with an aggressive clinical course. Integrated IHC and genomic analyses revealed no significant differences in phenotype or genomic profile between synchronous and metachronous UTUC-UBC cases. Despite molecular similarities, patients with synchronous UBC revealed significantly inferior PFS and OS compared to those with metachronous disease. These findings suggest that clinically synchronous tumors may harbor yet uncharacterized molecular features or microenvironmental influences that impact disease evolution.

Remarkably, our study underscores the complexity of monoclonality, while acknowledging that independent oncogenic events within a genetically primed urothelial field may give rise to distinct multifocal entities. Moreover, the discrepancy between clinical chronology and molecular evolution highlights the heterogeneity of multifocal tumor development.

## Figures and Tables

**Table 1:** IHC staining and mutational status rates in UTUC + sUBC vs. UTUC + mUBC.

IHC Markers	Total, *n*/N (%)	UTUC + sUBC, *n*/N (%)	UTUC + mUBC, *n*/N (%)	OR (95% CI)	*p*-Value
CK5/6					
Positive	20/43 (46.5)	6/14 (42.9)	14/29 (48.3)	1.24 (0.34–4.50)	0.739 ^a^
Negative	23/43 (53.5)	8/14 (57.1)	15/29 (51.7)		
CK20					
Positive	26/43 (60.5)	8/14 (57.1)	18/29 (62.1)	1.23 (0.34–4.50)	0.757 ^a^
Negative	17/43 (39.5)	6/14 (42.9)	11/29 (37.9)		
GATA3					
Positive	16/43 (37.2)	8/14 (57.1)	8/29 (27.6)	0.29 (0.08–1.09)	0.060 ^a^
Negative	27/43 (62.8)	6/14 (42.9)	21/29 (72.4)		
p53					
Wild-type	34/42 (81)	9/13 (69.2)	25/29 (86.2)	0.36 (0.07–1.75)	0.226 ^b^
Aberrant	8/42 (19)	4/13 (30.8)	4/29 (13.8)		
Luminal-basal phenotype					
Luminal	18/37 (48.6)	6/11(54.5)	12/26 (46.2)	1.40 (0.34–5.77)	0.641 ^a^
Basal	19/37 (51.4)	5/11 (45.5)	14/26 (53.8)		
**Gene Status**					
*FGFR3*					
Wild-type	22/42 (52.4)	8/14 (57.1)	14/28 (50)	1.33 (0.37–4.85)	0.662 ^a^
Mutated	20/42 (47.6)	6/14 (42.9)	14/28 (50)		
Specific mutations					
Exon 7 p.R248C	10/42 (23.8)	3/14 (21.4)	7/28 (25)		
Exon 7 p.S249C	7/42 (16.7)	1/14 (7.1)	6/28 (21.4)		
Exon 7 p.R248C + p.S249C	3/42 (7.1)	2/14 (14.3)	1/28 (3.6)		
*TERTp*					
Wild-type	19/41 (46.3)	4/13 (30.8)	15/28 (53.6)	0.385 (0.10–1.55)	0.173 ^b^
Mutated	22/41 (53.7)	9/13 (69.2)	13/28 (46.4)		
Specific mutations					
c.1-124G > A	16/41 (39)	7/13 (53.8)	9/28 (32.1)		
c.1-146G > A	6/41 (14.6)	2/13 (15.4)	4/28 (14.3)		
*RAS*					
Wild-type	39/43 (90.7)	13/14 (92.9)	26/29 (89.7)	1.50 (0.14–15.87)	0.607 ^b^
Mutated	4/43 (9.3)	1/14 (7.1)	3/29 (10.3)		
Specific mutations					
HRAS1 p.G13R	1/43 (2.3)	1/14 (7.1)	0		
KRAS1 p.G12D	1/43 (2.3)	0	1/29 (3.4)		
KRAS1 p.V14I	1/43 (2.3)	0	1/29 (3.4)		
HRAS2 p.Q61R + KRAS2 p.Q61P	1/43 (2.3)	0	1/29 (3.4)		

Data are presented as *n*/N (%), where n represents the number of cases with the feature and N represents the total number of evaluable samples for that specific variable. Valid percentages were calculated based on available data to account for missing values. ^a^ Chi-square test; ^b^ Fisher’s exact test. Abbreviations: IHC—immunohistochemical; n—number of patients; OR—odds ratio; UBC—urothelial bladder cancer; UTUC—upper tract urothelial carcinoma; UTUC + mUBC—UTUC with metachronous UBC; UTUC + sUBC—UTUC with synchronous UBC.

## Data Availability

The original contributions presented in this study are included in the article/Supplementary Materials. Further inquiries can be directed to the corresponding author.

## Supplementary Materials

The following supporting information can be downloaded at: https://www.mdpi.com/article/10.3390/cimb48040345/s1.

## Abbreviations

The following abbreviations are used in this manuscript:

UTUC	Upper tract urothelial carcinoma
UBC	Urothelial bladder cancer
RNU	Radical nephroureterectomy
UTUC + sUBC	Upper tract urothelial carcinoma with synchronous bladder cancer
UTUC + mUBC	Upper tract urothelial carcinoma with metachronous bladder cancer
FFPE	Formalin-fixed, paraffin-embedded
H&E	Hematoxylin and eosin
IHC	Immunohistochemical
CK	Cytokeratin
TERTp	TERT promoter
OS	Overall survival
PFS	Progression-free survival
IQR	Interquartile range
NMIBC	Non-muscle invasive bladder cancer
