# Supplementary material for: Differentiating Upper Tract Urothelial Carcinoma with Synchronous or Metachronous Bladder Cancer
Source: Curr Issues Mol Biol. 2026 Mar 26;48(4):345. doi: 10.3390/cimb48040345 (PMC13114334; doi:10.3390/cimb48040345)
Supplement: Supplementary file 1 [file cimb-48-00345-s001.zip › Supplementary File S1 - Material and Methods.pdf]

Supplementary Files S1:

Detailed experimental procedures

Tumour Specimens

Representative sections of all archived formalin-fixed, paraffin-embedded (FFPE) tumor samples from UTUC patients who underwent either radical nephroureterectomy or kidney-sparing surgery were examined. Sections were stained with hematoxylin and eosin (H&E) and reviewed by a specialized genitourinary pathologist. Tumour-containing tissue samples were staged according to the 2002 American Joint Committee on Cancer (AJCC) criteria and graded based on the 2004 WHO pathological grading system for malignant urothelial carcinoma.

DNA Extraction

Following histopathological assessment, genomic DNA was isolated from FFPE tissue in accordance with the manufacturer's instructions (GRS Genomic DNA Kit, GRiSP, Porto, Portugal). Tumour regions were manually microdissected using corresponding H&E-stained slides as a guide. The extracted DNA was then stored at -20 °C or at 4°C for immediate use.

Immunohistochemical Analysis

The expression of the proteins CK5/6, CK20, GATA3, and p53 was analyzed to classify clinically relevant tumour subtypes. Immunohistochemistry (IHC) was performed using the UltraVision™ Quanto Detection System HRP (REF: TL-125-QHL, Thermo Scientific, Waltham, MA, USA). Tissue sections were first deparaffinized and rehydrated, then subjected to heat-induced epitope retrieval at 90°C for 30 minutes using 10× Epitope Retrieval Solution (pH 9.0; Novocastra™, Ref. RE7119, Leica Biosystems, NuBlock, Germany). For p53 detection, antigen retrieval was carried out in 10 mM sodium citrate buffer (pH 6.0). Endogenous peroxidase activity was blocked using Ultravision™ hydrogen peroxide block (REF. TA-125-H202Q, Thermo Scientific, Fremont, CA, USA), and nonspecific binding was minimized with UltraVision™ protein block (REF. TA-125-PBQ, Thermo Scientific, Fremont, CA, USA).

Slides were incubated overnight at 4°C in a humidified chamber with monoclonal antibodies against CK5/6 (1:100, clone D5/16 B4, Dako) and CK20 (1:100, clone Ks20.8, Dako). For p53, sections were incubated at room temperature for 60 minutes with anti-p53 antibody (1:600, clone DO-7, Leica). Following PBS washes, slides were treated with HRP Polymer Quanto (REF. TL-125-QPH) and visualized using 3% diaminobenzidine (DAB, REF. TA-004-QHCX) together with DAB Quanto Substrate (REF. TA-125-QHSX). CK5/6 staining was additionally visualized with HIGHDEF® Red IHC Chromogen (HRP, REF. ADI-950-210-0030, Enzo Life Sciences, Farmingdale, NY, USA). Finally, sections were counterstained with Gill's hematoxylin, cleared, and mounted.

GATA3 immunostaining (ready-to-use, clone L50-823, Master-inVitro Diagnóstica, Sevilla, Spain) was carried out on a Ventana Benchmark XT automated platform, with chromogenic development performed using the OptiView DABv3 detection kit according to the manufacturer's guidelines.

Expression of CK5/6, CK20, and GATA3 was assessed semi-quantitatively based on staining intensity (0 = absent; 1 = faint; 2 = moderate; 3 = strong) and the percentage of positive tumour cells (<5% = 0; 5–25% = 1; 25–50% = 2; 50–75% = 3; >75% = 4). The immunoreactive score (IRS) was calculated by multiplying the intensity and proportion scores. Cut-off values for positivity were set using the median IRS values (CK5/6 > 3, CK20 > 6, GATA3 > 8). p53 expression was categorized as wild-type (1–49% nuclear staining) or aberrant (null phenotype: 0%; 50–99%; or diffuse overexpression: 100%).

Tumours were classified as luminal-like if CK20+ or GATA3+/CK5/6-, and basal-like if CK20- or GATA3-/CK5/6+.

Targeted Sequencing Genomic Characterization

Genomic profiling of UTUC was conducted by targeting mutations commonly observed in urothelial carcinoma, focusing on *TERT* promoter (*TERTp*), *FGFR3*, *RAS* family genes (*HRAS*, *KRAS*, *NRAS*), and *TP53*.

Hotspot mutations in the *TERT* promoter (NM_198253; positions -124 and -146 relative to the transcription start site) and in *FGFR3* exon 7 (NM_000142; codons 248 and 249) were detected using quantitative real-time PCR (QuantStudio™ 5, Applied Biosystems, Waltham, MA, USA), employing primers and probes provided by the Uromonitor® kit (U-monitor, Porto, Portugal) according to the manufacturer's protocol.

Mutational analysis of *TP53* (exons 5–9; NM_000546), *HRAS* (codons 12, 13, 61; NM_005343), and *KRAS* (codons 12, 13, 61; NM_004985) was performed by Sanger sequencing, while only codon 61 was analyzed for *NRAS* (NM_002524).

Genomic DNA (25–50 ng) was amplified using the QIAGEN multiplex PCR kit for *TP53* and MyTaq HS Mix 2X (Bioline) for *RAS* genes, following manufacturer instructions. *NRAS* fragments were amplified with an annealing temperature of 60°C, whereas *TP53*, *HRAS*, and *KRAS* were amplified separately using touchdown PCR. Successful amplification was confirmed on 1% agarose gels (GRS Agarose LE, GRiSP, Porto, Portugal). PCR products were then purified with Exonuclease I and Shrimp Alkaline Phosphatase (Thermo Scientific, Vilnius, Lithuania) and sequenced using the Big Dye Terminator v3.1 Cycle Sequencing Kit (Applied Biosystems, Portsmouth, NH, USA). Following precipitation, sequencing reactions were resolved by capillary electrophoresis and analyzed using an ABI PRISM 3100 Genetic Analyzer (Perkin-Elmer, Foster City, CA, USA). All detected mutations were independently confirmed through repeat PCR analysis.
