# Supplementary material for: Differentiating Upper Tract Urothelial Carcinoma with Synchronous or Metachronous Bladder Cancer
Source: Curr Issues Mol Biol. 2026 Mar 26;48(4):345. doi: 10.3390/cimb48040345 (PMC13114334; doi:10.3390/cimb48040345)
Supplement: Supplementary file 1 [file cimb-48-00345-s001.zip › Supplementary File S2.pdf]

Supplementary Files S2

Figures and Tables

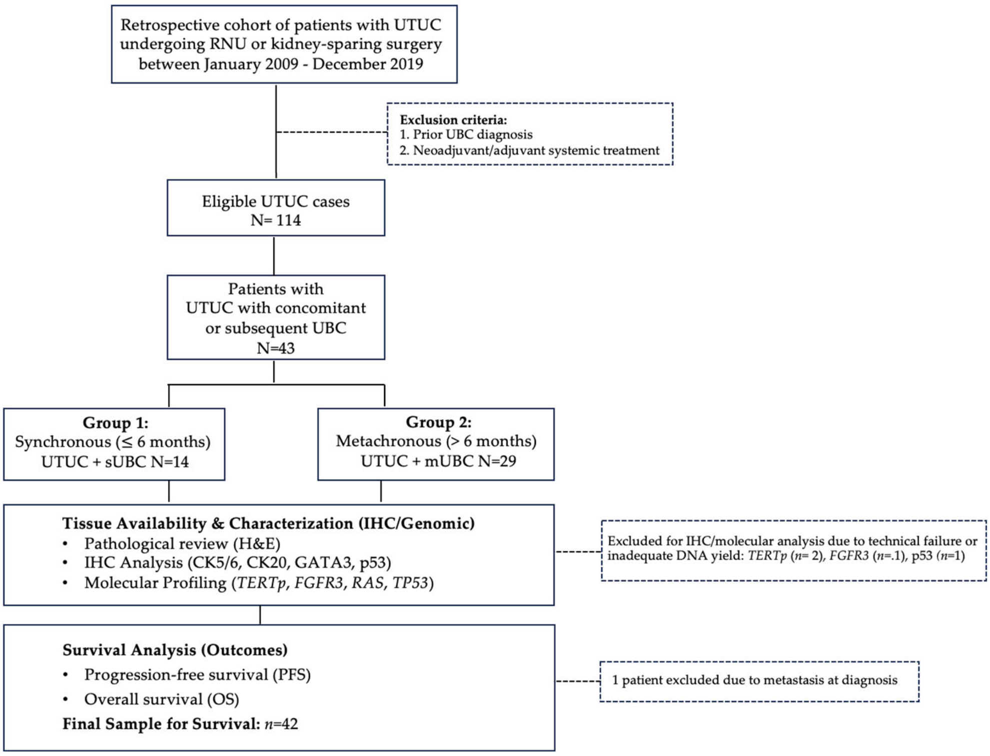

Figure S1. Flowchart of patient selection and study design. Diagram illustrating the retrospective cohort screening of patients with UTUC between January 2009 and December 2019. The chart details the exclusion criteria and the final stratification into two subgroups: synchronous (UTUC+sUBC) and metachronous (UTUC+mUBC) bladder cancer. The bottom sections summarize the integrated workflow for immunohistochemical profiling, genomic characterization, and survival analysis (OS and PFS).

Abbreviations: CK – cytokeratin; H&E – hematoxylin and eosin; IHC – immunohistochemical; N—number of patients; OS – overall survival; PFS – progression-free survival; RNU – radical nephroureterectomy; UBC—urothelial bladder cancer; UTUC—upper tract urothelial carcinoma; UTUC+mUBC – UTUC with metachronous UBC; UTUC+sUBC – UTUC with synchronous UBC.

Table S1. Baseline clinicopathologic characteristics of UTUC+sUBC vs UTUC+mUBC patients.

Baseline Characteristics	Total, *n*/N (%)	UTUC+sUBC, *n*/N (%)	UTUC+mUBC, *n*/N (%)	*p*-value
**Number of patients** (n / %)	43 (100)	14 (32.6)	29 (67.4)	
**Age** (median, IQR, years)	77 (67-82)	77 (71-81)	75 (64-84)	0.969[a]
**Gender**				
Male	30/43 (69.8)	11/14 (78.6)	19/29 (65.5)	0.491[c]
Female	13/43 (30.2)	3/14 (21.4)	10/29 (34.5)	
**Smoking**				
Yes	18/33 (54.5)	7/11 (63.6)	11/22 (50)	0.458[b]
No	15/33 (45.5)	4/11 (36.4)	11/22 (50)	
**Hydronephrosis**				
Yes	20/41(48.8)	6/14 (42.9)	14/27 (51.9)	0.585[b]
No	21/41(51.2)	8/14 (57.1)	13/27 (48.1)	
**Tumor location**				
Renal pelvis	21/43 (48.8)	7/14 (50)	14/29 (48.3)	0.970[c]
Ureter	15/43 (34.9)	5/14 (35.7)	10/29 (34.5)	
Both	7/43 (16.3)	2/14 (14.3)	5/29 (17.2)	
**Surgical procedure**				
Nephroureterectomy	37/43 (86)	12/14 (85.7)	25/29 (86.2)	0.649[c]
Kidney-sparing approach	6/43 (14)	2/14 (14.3)	4/29 (13.8)	
**Histological subtype**				
Pure UC	39/43 (90.7)	14/14 (100)	25/29 (86.2)	0.286[c]
Non-pure UC	4/43 (9.3)	0	4/29 (13.8)	
**Lymphadenectomy**				
Yes	12/42 (28.6)	3/13 (23.1)	9/29(31)	0.722[c]
No	30/42 (71.4)	10/13 (76.9)	20/29 (69)	
**Tumor size, cm**				
≤ 2	6/43 (14)	3/14 (21.4)	3/29 (10.3)	0.373[c]
> 2	37/43 (86)	11/14 (78.6)	26/29 (89.7)	
**Multifocality**				
Yes	6/43 (14)	3/14 (21.4)	3/29 (10.3)	0.373[c]
No	37/43 (86)	11/14 (78.6)	26/29 (89.7)	
**Tumor grade**				
Low-grade	3/43 (7)	1/14 (7.1)	2/29 (6.9)	0.704[c]
High-grade	40/43 (93)	13/14 (92.9)	27/29 (93.1)	
**Lymphovascular invasion**				
Yes	11/43 (25.6)	4/14 (28.6)	7/29 (24.1)	0.515[c]
No	32/43 (74.4)	10/14 (71.4)	22/29 (75.9)	
**Carcinoma *in situ***				
Yes	7/43 (16.3)	4/14 (28.6)	3/29 (10.3)	0.190[c]
No	36/43 (83.7)	10/14 (71.4)	26/29 (89.7)	
**AJCC staging***				
0is + 0a + I	13/42 (31)	4/13 (30.8)	9/29 (31)	0.553[c]
II + III + IV	29/42 (69)	10/13 (76.9)	19/29 (65.5)	
**Lymph node involvement**				
Yes	1/12 (8.3)	4/14 (28.6)	7/29 (24.1)	0.515[c]
No	11/12 (91.7)	10/14 (71.4)	22/29 (75.9)	

**Metastasis at diagnosis**				
Yes	1/43 (2.3)	1/14 (7.1)	0	0.674[c]
No	42/43 (97.7)	13/14 (92.9)	29/29 (100)	

Data are presented as *n*/N (%), where n represents the number of cases with the feature and N represents the total number of evaluable samples for that specific variable. Valid percentages were calculated based on available data to account for missing values; * excluded patients with metastasis at diagnosis and locally advanced unresectable disease; [a]Mann-Whitney U-test; [b]Chi-square test; [c]Fisher's exact test; Abbreviations: AJCC—American Joint Committee on Cancer; cm—centimeter; ECOG PS—Eastern Cooperative Oncology Group Performance Status; n—number of patients; UBC—urothelial bladder cancer; UC—urothelial carcinoma; UTUC—upper tract urothelial carcinoma; UTUC+mUBC – UTUC with metachronous UBC; UTUC+sUBC – UTUC with synchronous UBC.

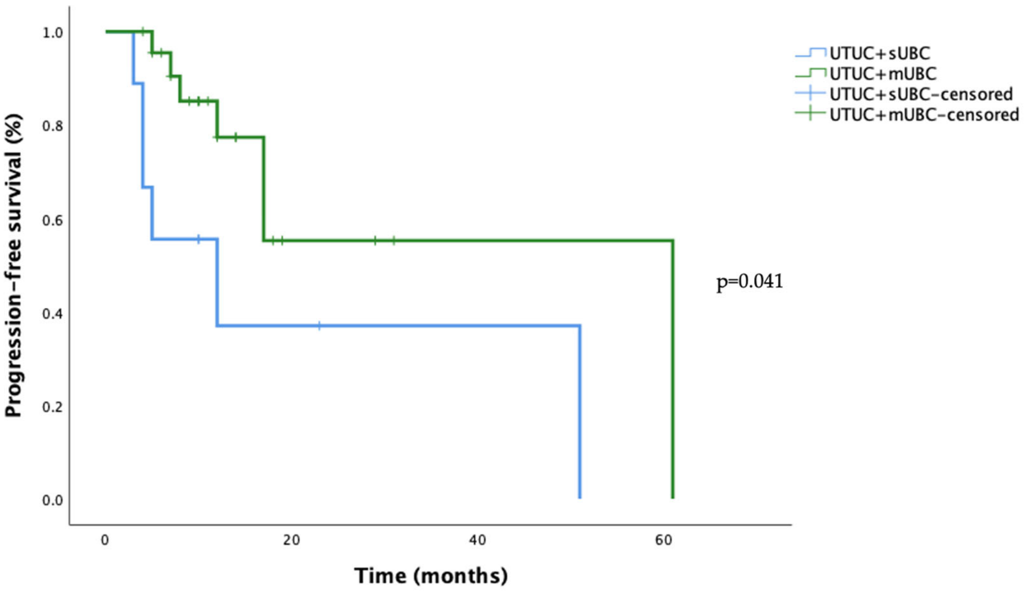

Figure S2. Sensitivity analysis of progression-free survival (PFS) excluding intravesical recurrences. Kaplan–Meier curves comparing PFS between patients with UTUC+sUBC (blue line) and those with UTUC+mUBC (green line), excluding all intravesical events from the PFS definition to mitigate potential circularity. Abbreviations: UBC—urothelial bladder cancer; UTUC—upper tract urothelial carcinoma; UTUC+mUBC – UTUC with metachronous UBC; UTUC+sUBC – UTUC with synchronous UBC; Log-rank test, statistical significance *p*-value < 0.05. This figure is original and was created by the authors for this study.

Table S2. 6-month landmark sensitivity analysis for progression-free Survival (PFS) and overall survival (OS).

**Parameter**	**UTUC + sUBC (*n*=13)**	**UTUC + mUBC (*n*=29)**	***p*-value**
**Total Events (N)**			
Progression-free Survival (PFS)	10	13	0.029
Overall Survival (OS)	8	11	0.016
**Early Events/Censoring (0-6 months)**			
Events (PFS/OS)	2/1	0/0	
Censored observations (PFS/OS)	0/0	0/0	
**Landmark Analysis (at 6 months)**			
Eligible patients at Landmark (N)	11	29	
Events after Landmark (PFS)	8	13	0.047
Events after Landmark (OS)	7	11	0.038

This table presents the distribution of events and censored observations used for the landmark analysis. In the UTUC+sUBC group (n=13), two patients experienced a PFS event (progression or death) within the first 6 months, while one patient died (OS event) during the same period. Consequently, 11 patients were eligible for the PFS landmark analysis. In the UTUC+mUBC group (n=29), no early events or censoring occurred, and all patients remained eligible at the 6-month mark. *p*-values refer to between-group comparison of events occurring after the 6-month landmark using the log-rank test. This table is original and was created by the authors for this study. Abbreviations: UBC—urothelial bladder cancer; UTUC—upper tract urothelial carcinoma; UTUC+mUBC – UTUC with metachronous UBC; UTUC+sUBC – UTUC with synchronous UBC; Log-rank test, statistical significance p-value < 0.05.
